# A Novel Vaccine Selection Decision-Making Model (VSDMM) for COVID-19

**DOI:** 10.3390/vaccines9070718

**Published:** 2021-07-01

**Authors:** Sayed F. Abdelwahab, Usama H. Issa, Hossam M. Ashour

**Affiliations:** 1 Department of Pharmaceutics and Industrial Pharmacy, College of Pharmacy, Taif University, Taif 21944, Saudi Arabia; s.fekry@tu.edu.sa; 2 Department of Civil Engineering, College of Engineering, Taif University, Taif 21944, Saudi Arabia; u.issa@tu.edu.sa; 3 Department of Integrative Biology, College of Arts and Sciences, University of South Florida, St. Petersburg, FL 33701, USA

**Keywords:** analytic hierarchy process, COVID-19, decision-making model, vaccines

## Abstract

Selecting a vaccine for fighting a pandemic is one of the serious issues in healthcare. Novel decision models for vaccine selection need to be developed. In this study, a novel vaccine selection decision-making model (VSDMM) was proposed and developed, based on the analytic hierarchy process (AHP) technique, which assesses many alternatives (vaccines) using multi-criteria to support decision making. To feed data to the VSDMM, six coronavirus disease-19 (COVID-19) vaccines were selected in a case study to highlight the applicability of the proposed model. Each vaccine was compared to the others with respect to six criteria and all criteria were compared to calculate the relative weights. The proposed criteria include (1) vaccine availability; (2) vaccine formula; (3) vaccine efficacy; (4) vaccine-related side effects; (5) cost savings, and (6) host-related factors. Using the selected criteria, experts responded to questions and currently available COVID-19 vaccines were ranked according to their weight in the model. A sensitivity analysis was introduced to assess the model robustness and the impacts of changing criteria weights on the results. The VSDMM is flexible in terms of its ability to accept more vaccine alternatives and/or more criteria. It could also be applied to other current or future pandemics/epidemics in the world. In conclusion, this is the first report to propose a VSDMM for selecting the most suitable vaccines in pandemic/epidemic situations or any other situations in which vaccine selection and usage may be deemed necessary.

## 1. Introduction

Decision making in healthcare includes a complex set of pragmatic interactions among many stakeholders [1]. Limited mathematical models or techniques are currently used to support the selection of a suitable vaccine for fighting pandemics/epidemics. There is a lack of literature regarding factors that could lead to the acceptance or rejection of a vaccine among vaccine alternatives. The analytic hierarchy process (AHP) was introduced in 1980 [2] to help resolve decision-making issues and prioritize decision alternatives. It has been widely used in decision-making systems in different fields since then to help solve various problems [3,4,5,6,7,8,9]. Recently, researchers used AHP in healthcare and medical sectors to support decision-making. For example, health intervention options were evaluated and analyzed for basic scoring using AHP [10]. Another decision problem for the aeroengine health assessment was supported using a process called fuzzy-AHP, in which a three-step evaluation model was proposed using eleven criteria for presenting advantages and disadvantages of the alternatives [11]. In addition, a model that makes use of different clustering algorithms and applies the AHP method was developed to facilitate analysis and auditing of suspicious claims data from healthcare providers [12]. The same process, fuzzy-AHP, was used to examine the factors of service quality in the healthcare sector in Turkey [13]. AHP was used in the implementation of an electronic service quality framework for healthcare services via internet by using combined multiple criteria in decision making [14]. The AHP technique was also utilized to construct a clinical decision support system [15]. Moreover, the AHP technique could be applied in risk assessment to determine the amount of risk associated with outsourcing logistics to cope with various hazards and uncertainties in the pharmaceutical supply chain [16]. In addition, to examine the scalability of electronic health records systems, the AHP approach was paired with a discrete-event simulation tool [17].

A prioritization approach for health technology assessment was developed based on AHP and results highlighted the importance of treatment effectiveness, patient safety, and societal aspects in the decision-making process for best treatment alternatives in dialysis [1] and Korean medicine [18]. 

AHP uses actual measures, such as price, counts, or subjective opinions, as inputs into mathematical matrices [19]. The AHP technique can help decision makers reach a logical ranking order of alternatives in a shorter time and using less information than is typically needed. The outputs include ratio scales and consistency indices derived by computing eigen values and eigen vectors. As a decision-making framework for the assessment of interrelationships amongst multi-criteria, AHP assumes a unidirectional hierarchical relationship among decision levels [20,21]. The examination of the interaction among multiple criteria is a more practical approach given that connections between criteria often exist [22].

Importantly, there is a shortage in the literature in factors and mechanisms for the selection of a vaccine among vaccine alternatives. Therefore, the development of a discrete choice model to investigate the different criteria for vaccine selection among available alternatives is critical. In this study, a vaccine selection decision-making model (VSDMM) was developed. Six criteria were identified to support vaccine selection: (1) vaccine availability which depends on the accessibility to the vaccine and its manufacturing location; (2) vaccine formula, which includes recombinant, subunit, inactivated, viral vector, DNA, and RNA; (3) vaccine efficacy [23], which can lead to an increase in the value of the vaccine; (4) vaccine-related side effects, which may be major or minor and their prevalence (In this regard, mild and infrequent side effects will lead to a higher value for the vaccine); (5) cost savings, which include vaccine price, transportation costs, and storage costs, and (6) host-related factors, which include age, immune status, body mass index, and comorbidities, including heart diseases, diabetes, obesity, hypertension, and immune disorders. In this regard, a vaccine that is suitable for all ages and for patients with different comorbidities will have a higher value. 

Using this model, decision makers can select the most relevant vaccine for each situation. It is important to note that this is a proof-of-principal study that aims to highlight the applicability of this model to the decision-making process for COVID-19 vaccine selection.

## 2. Methods

The proposed methodology included the following five phases: (1) identify criteria that control vaccine selection for a certain pandemic/epidemic; (2) propose and develop a VSDMM that can support vaccine selection from the available alternatives; (3) select the case study and alternative vaccines (six vaccines in the current case study); (4) collect data to feed and test the model, and (5) apply the VSDMM model on the case study and analyze the results. The proposed criteria included vaccine availability, vaccine formula, vaccine efficacy, vaccine-related side effects, cost savings, and host-related factors. 

### 2.1. Vaccine Selection Decision-Making Model (VSDMM)

We developed an AHP-based decision model that can provide support for the selection of the most suitable vaccine from many available vaccine alternatives for the COVID-19 pandemic. The problem was organized in hierarchical terms then the relative importance of each criterion was determined by pairwise comparison to other criteria. The first level of questions to decision makers included questions about the importance of a criterion as compared to another criterion regardless of the nature of the vaccine. The second level of questions to decision makers included questions about the importance of a particular criterion for one vaccine as compared to the other vaccine. The model comprised one matrix in its first level (6 × 6) and six matrices in the second level (6 × 6). Comparisons were performed by utilizing the preference scale [19]. According to the protocol, the decision maker has to choose a defined number from 1 to 9 through pairwise comparisons of the elements. We employed a nine-point scale in which a value of 1 denoted “equally important”, 3 denoted “somewhat more important”, 5 denoted “much more important”, 7 denoted “very much more important”, and 9 denoted “absolutely important”. The consistency ratio (CR) was a measure for the cognitive effort in the decision. CR has been calculated as the consistency index (CI) divided by the relative importance (RI), also known as the eigen vector [2].

### 2.2. Case Study Selection for COVID-19 Vaccine Alternatives

In this study, we proposed a hesitant fuzzy AHP method to help health policymakers evaluate the importance of intervention strategy alternatives for the COVID-19 pandemic [24]. Developers of leading COVID-19 candidate vaccines continue to apply for authorizations for their vaccines [25]. Four vaccines, two mRNA-based vaccines (Pfizer-BioNTech and NIH-Moderna) and two non-replicating viral vector-based vaccines (Oxford-AstraZeneca and Janssen) have been made available through national health services (NHS) [26]. We included six vaccines in this study. The selected vaccines were the vaccines of Pfizer BioNTech [27], NIH-Moderna [28], AstraZeneca [29,30], Sinopharm [31], Sputnik V [32], and Janssen Research & Development [33]. The model can accommodate any other future vaccines. Further information about these vaccines can be found in the respective references [27,28,29,30,31,32,33]. 

### 2.3. Model Application 

Selecting a vaccine is a multiple criteria decision-making (MCDM) problem with potentially competing criteria. The data for feeding and testing the model were collected through brainstorming sessions which is a typical documentation technique for collecting data. Two brainstorming sessions were conducted with two expert scientists in virology and vaccines. Respondent 1 (first session) was a professor of vaccine immunology and clinical microbiology, whereas respondent 2 (second session) was a professor of virology. The first step was to determine the importance and weight of each criterion. In the first level, the model was fed with values of the pairwise comparison matrices for the six identified criteria. Using the decision model, the relative weights of the criteria were calculated for the first and second sessions, as shown in Table 1. The consistency ratios (CRs) were 9.5% and 7.9%, indicating that the matrix was consistent (CR < 10%) in accordance to what was previously reported [2]. For the second level, six matrices were completed for session 1 (Table 2) and six were also completed for session 2 (Table 3). The respondents were asked various questions including questions about the importance of selecting vaccine 01 versus vaccine 02 with respect to all criteria, such as the availability criterion and formula criterion. As indicated previously, six criteria that can affect the decision-making process for vaccine selection were used when comparing the vaccines. 

### 2.4. Sensitivity Analysis

Sensitivity analysis was conducted using the VSDMM for the data from respondent 1 as an example to showcase the effects of weight changes of the selected criteria on the model results. The weight of each criterion was increased or decreased by a value of 1 (to the next higher or lower weight values). The impact of this change was analyzed.

## 3. Results

After identifying the vaccine alternatives and their respective characteristics, the model was applied based on the selections of the respondents, which were based on their opinions and experience. The responses of the two selected experts that were fed into the model are shown in Table 1, Table 2 and Table 3. 

**Figure 1:**
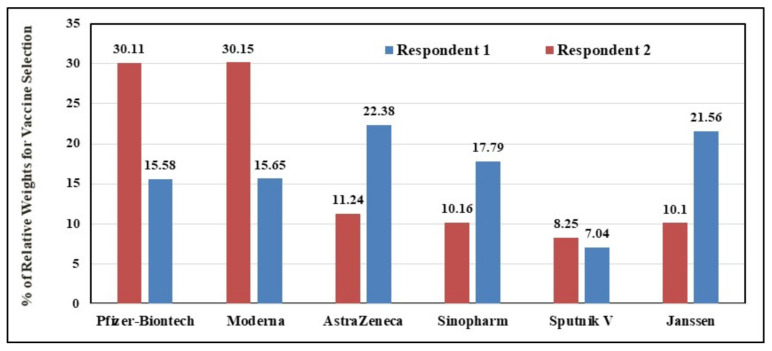
Percentage of Relative Weights for Vaccine Selection.

Based on the responses of the two experts, the relative weights for all vaccines were summarized in Table 4 and Figure 1, in accordance with the six comparison criteria. As evident from Table 4, there were areas of agreement and areas of disagreement between the two respondents. The first respondent’s selections indicated a preference for the AstraZenca and Janssen vaccines over the other types. Selections for the second respondent indicated a preference for the Pfizer-BioNTech and NIH-Moderna vaccines (Figure 1).

In addition, the first respondent preferred AstraZeneca (22.38%) followed by Janssen (21.56%) while the Sputnik V vaccine came in the last place (7.04%) (Figure 1). On the other hand, the second respondent’s selections indicated preference for the NIH-Moderna vaccine followed by the Pfizer-BioNTech vaccine (30.15% and 30.11%, respectively), whereas the Sputnik V vaccine came in the last rank (8.25%). Selections of the first respondent indicated clear preference for AstraZeneca and Janssen due to their high efficacy and availability (Table 4). On the other hand, the second respondent’s selections indicated a preference for Pfizer-BioNTech and NIH-Moderna, mainly due to the criteria of availability and formula.

### Sensitivity Analysis

As shown in Table 5, there were no significant changes in the model outcomes by modifying weights of the criteria. The maximum change did not exceed 3.77% (efficacy criterion upon selecting the Sinopharm vaccine) and the rankings of the vaccines were not affected in all cases.

## 4. Discussion

COVID-19 continues to spread, rapidly challenging governments and decision makers in different parts of the world [34]. This global spread has created an urgent need for safe and effective vaccines. External factors that impact the process of vaccine approval could significantly undermine public health efforts to promote the use of the vaccine as a way to end the devastating pandemic [35]. Vaccine recommendation and wide-scale acceptance require consideration of different social and human factors [36]. It has been shown that COVID-19 vaccine allocation for older adults (>60 years) led to the highest relative reduction in deaths, irrespective of vaccine efficacy [37]. There were significant demographic and geographical disparities in COVID-19 vaccine acceptance rates in the U.S. [38]. A study on vaccination behaviors of nurses revealed that COVID-19-related work demands of nurses were associated with higher work stress, and thus a stronger intention to receive COVID-19 vaccination [39]. 

Among the six vaccine selections included in this case study, mRNA-based vaccines (Pfizer-BioNTech and NIH-Moderna) and non-replicating viral vector-based vaccines (Oxford-AstraZeneca and Janssen) were considered [26]. A health technology assessment approach based on AHP showed that decision makers gave priority to treatment effectiveness and patient safety when deciding on best treatment alternatives in dialysis [1]. An AHP-based approach was used to evaluate priority for the standardization of traditional medicine and included variables such as technology evolution, political importance, and economic efficiency [18]. Finally, a fuzzy AHP-VIKOR (f-visekriterijumska optimizacija i kompromisno resenje) method was proposed to help decision makers prioritize intervention strategies in influenza and for influenza vaccine selection using a different set of criteria [40].

It is best for the decision makers to consult specialists in vaccines, in addition to specialists in other fields such as cost analysis and social aspects. The number of experts needed to make a good decision depends on the particulars of the model and other relevant conditions. Importantly, media coverage can certainly affect the people’s selection of vaccine but should not affect the conclusions of decision makers.

The AHP technique has many advantages, such as flexibility and ease of use, through using different variables in selection and comparing alternatives based on multiple criteria. Moreover, the model is suitable for use in different countries with slight modifications that are based on selection criteria and available alternatives. On the other hand, most of the mathematical models have limitations. Two limitations appeared during the application of the proposed model in the study. The first arose when the consistency ratio (CR) of the results exceeded 10%, which could negatively impact the accuracy. This issue can sometimes lead the model user to ask respondents to reconsider some of their selections, which may not always be acceptable to the respondents. Another limitation is that there can sometimes be a need to merge some of the criteria that are close to each other, which cannot be simply achieved by decreasing the number of alternatives.

In summary, the evaluation and selection of suitable vaccines for epidemics/pandemics can be complex given that each vaccine selection has advantages and disadvantages. In addition, the criteria used to compare vaccines are controlled by different factors. An AHP-based model for decision-making was developed to support vaccine selection and was applied on a COVID-19 vaccine case study (model) as an example that can be expanded upon for the current pandemic and future pandemics. Results obtained from sensitivity analysis indicated that the ranking order of alternatives remained consistent, even when the weights of the criteria were slightly changed. Decision makers can always modify or add to the comparison criteria or to the number of vaccine alternatives. The decision can also be made based on inputs from numerous decision makers (and not just two). These advantages can give decision makers the ability to select the best vaccine that suits their specific situation using the VSDMM. 

## Figures and Tables

**Table 1:** Comparison matrix of model input for the six comparison criteria (Respondents 1 and 2).

Criterion	Availability		Formula		Efficacy		Side Effects		Cost		Host Factors	
	Resp.1	Resp.2	Resp.1	Resp.2	Resp.1	Resp.2	Resp.1	Resp.2	Resp.1	Resp.2	Resp.1	Resp.2
Availability	1	1	0.25	0.2	0.142	0.142	2	1	7	7	4	1
Formula	4	5	1	1	0.167	0.5	5	3	6	8	4	2
Efficacy	7	7	6	2	1	1	8	2	9	8	8	3
Side Effects	0.5	1	0.2	0.333	0.125	0.5	1	1	3	4	1	0.333
Cost	0.142	0.142	0.167	0.125	0.111	0.125	0.333	0.25	1	1	0.333	0.25
Host Factors	0.25	1	0.25	0.5	0.125	0.333	1	3	3	4	1	1

CR (Respondent 1) = 9.5%, CR (Respondent 2) = 7.9%, 1 = Equally important, 3 = Somewhat more important, 5 = Much more important, 7 = Very much more important, 9 = Absolutely important. The grey color indicates the selection of the respondents (Resp.1 and Resp.2). The white color indicates the inverse value of the selection of the respondent. The pale blue color indicates a value of 1 (equally important) when comparing an alternative to itself.

**Table 2:** Comparison matrices of model inputs for vaccines (Respondent 1).

**Comparison Matrix for Vaccines with Respect to: Availability (CR = 4.1%)**						
**Vaccine**	**Pfizer-BioNTech**	**Moderna**	**AstraZeneca**	**Sinopharm**	**Sputnik V**	**Janssen**
Pfizer-BioNTech	1	1	1	1	3	1
Moderna	1	1	1	1	3	1
AstraZeneca	1	1	1	3	3	1
Sinopharm	1	1	0.333	1	3	0.333
Sputnik V	0.333	0.333	0.333	0.333	1	0.5
Janssen	1	1	1	3	2	1
**Comparison Matrix for Vaccines with Respect to: Formula (CR = 4.9%)**						
**Vaccine**	**Pfizer-BioNTech**	**Moderna**	**AstraZeneca**	**Sinopharm**	**Sputnik V**	**Janssen**
Pfizer-BioNTech	1	1	0.333	0.333	3	0.333
Moderna	1	1	0.333	0.333	3	0.333
AstraZeneca	3	3	1	0.5	3	1
Sinopharm	3	3	2	1	4	2
Sputnik V	0.333	0.333	0.333	0.25	1	0.5
Janssen	3	3	1	0.5	2	1
**Comparison Matrix for Vaccines with Respect to: Efficacy (CR = 7.9%)**						
**Vaccine**	**Pfizer-BioNTech**	**Moderna**	**AstraZeneca**	**Sinopharm**	**Sputnik V**	**Janssen**
Pfizer-BioNTech	1	1	0.5	4	5	0.5
Moderna	1	1	0.5	4	5	0.5
AstraZeneca	2	2	1	4	3	1
Sinopharm	0.25	0.25	0.25	1	4	0.333
Sputnik V	0.2	0.2	0.333	0.25	1	0.333
Janssen	2	2	1	3	3	1
**Comparison Matrix for Vaccines with Respect to: Side Effects (CR = 4.7%)**						
**Vaccine**	**Pfizer-BioNTech**	**Moderna**	**AstraZeneca**	**Sinopharm**	**Sputnik V**	**Janssen**
Pfizer-BioNTech	1	1	2	0.25	3	3
Moderna	1	1	3	0.25	3	2
AstraZeneca	0.5	0.333	1	0.333	1	1
Sinopharm	4	4	3	1	4	3
Sputnik V	0.333	0.333	1	0.25	1	0.5
Janssen	0.333	0.5	1	0.333	2	1
**Comparison Matrix for Vaccines with Respect to: Cost (CR = 4.3%)**						
**Vaccine**	**Pfizer-BioNTech**	**Moderna**	**AstraZeneca**	**Sinopharm**	**Sputnik V**	**Janssen**
Pfizer-BioNTech	1	0.333	0.333	0.2	0.333	0.333
Moderna	3	1	0.333	0.2	0.333	0.333
AstraZeneca	3	3	1	0.333	0.5	1
Sinopharm	5	5	3	1	1	3
Sputnik V	3	3	2	1	1	2
Janssen	3	3	1	0.333	0.5	1
**Comparison Matrix for Vaccines with Respect to: Host Factors (CR = 4.7%)**						
**Vaccine**	**Pfizer-BioNTech**	**Moderna**	**AstraZeneca**	**Sinopharm**	**Sputnik V**	**Janssen**
Pfizer-BioNTech	1	1	1	0.5	0.333	1
Moderna	1	1	1	0.5	0.333	1
AstraZeneca	1	1	1	0.333	1	1
Sinopharm	2	2	3	1	3	3
Sputnik V	3	3	1	0.333	1	1
Janssen	1	1	1	0.333	1	1

1 = Equally important, 3 = Somewhat more important, 5 = Much more important, 7 = Very much more important, 9 = Absolutely important. The grey color indicates the selection of the respondent. The white color indicates the inverse value of the selection of the respondent. The pale blue color indicates a value of 1 (equally important) when comparing an alternative to itself.

**Table 3:** Comparison matrices of model inputs for vaccines (Respondent 2).

**Comparison Matrix for Vaccines with Respect to: Availability (CR = 7%)**						
**Vaccine**	**Pfizer-BioNTech**	**Moderna**	**AstraZeneca**	**Sinopharm**	**Sputnik V**	**Janssen**
Pfizer-BioNTech	1	1	7	3	7	7
Moderna	1	1	7	3	7	7
AstraZeneca	0.142	0.142	1	2	2	2
Sinopharm	0.333	0.333	0.5	1	2	0.5
Sputnik V	0.142	0.142	0.5	0.5	1	1
Janssen	0.142	0.142	0.5	2	1	1
**Comparison Matrix for Vaccines with Respect to: Formula (CR = 2.60%)**						
**Vaccine**	**Pfizer-BioNTech**	**Moderna**	**AstraZeneca**	**Sinopharm**	**Sputnik V**	**Janssen**
Pfizer-BioNTech	1	1	5	3	5	5
Moderna	1	1	5	3	5	5
AstraZeneca	0.2	0.2	1	0.333	1	1
Sinopharm	0.333	0.333	3	1	2	3
Sputnik V	0.2	0.2	1	0.5	1	3
Janssen	0.2	0.2	1	0.333	0.333	1
**Comparison Matrix for Vaccines with Respect to: Efficacy (CR = 5.70%)**						
**Vaccine**	**Pfizer-BioNTech**	**Moderna**	**AstraZeneca**	**Sinopharm**	**Sputnik V**	**Janssen**
Pfizer-BioNTech	1	1	3	7	3	3
Moderna	1	1	3	7	3	3
AstraZeneca	0.333	0.333	1	5	3	2
Sinopharm	0.142	0.142	0.2	1	0.333	0.2
Sputnik V	0.333	0.333	0.333	3	1	0.25
Janssen	0.333	0.333	0.5	5	4	1
**Comparison Matrix for Vaccines with Respect to: Side Effects (CR = 3.33%)**						
**Vaccine**	**Pfizer-BioNTech**	**Moderna**	**AstraZeneca**	**Sinopharm**	**Sputnik V**	**Janssen**
Pfizer-BioNTech	1	1	3	1	3	3
Moderna	1	1	3	1	3	3
AstraZeneca	0.333	0.333	1	1	1	1
Sinopharm	1	1	1	1	1	1
Sputnik V	0.333	0.333	1	1	1	1
Janssen	0.333	0.333	1	1	1	1
**Comparison Matrix for Vaccines with Respect to: Cost (CR = 9.30%)**						
**Vaccine**	**Pfizer-BioNTech**	**Moderna**	**AstraZeneca**	**Sinopharm**	**Sputnik V**	**Janssen**
Pfizer-BioNTech	1	1	0.2	0.5	0.5	0.5
Moderna	1	1	0.5	0.5	0.5	0.5
AstraZeneca	5	2	1	7	1	1
Sinopharm	2	2	0.142	1	0.333	0.142
Sputnik V	2	2	1	3	1	1
Janssen	2	2	1	7	1	1
**Comparison Matrix for Vaccines with Respect to: Host Factors (CR =3.35%)**						
**Vaccine**	**Pfizer-BioNTech**	**Moderna**	**AstraZeneca**	**Sinopharm**	**Sputnik V**	**Janssen**
Pfizer-BioNTech	1	1	3	3	3	3
Moderna	1	1	3	3	3	3
AstraZeneca	0.333	0.333	1	0.333	1	1
Sinopharm	0.333	0.333	3	1	3	3
Sputnik V	0.333	0.333	1	0.333	1	1
Janssen	0.333	0.333	1	0.333	1	1

1 = Equally important, 3 = Somewhat more important, 5 = Much more important, 7 = Very much more important, 9 = Absolutely important. The grey color indicates the selection of the respondent. The white color indicates the inverse value of the selection of the respondent. The pale blue color indicates a value of 1 (equally important) when comparing an alternative to itself.

**Table 4:** Relative weights of vaccines based on the six comparison criteria.

Vaccine	Respondent	Availability	Formula	Efficacy	Side Effects	Cost	Host Factors
Pfizer-BioNTech	Respondent 1	18.4	10.2	17.8	17.7	5.3	11.5
	Respondent 2	35.6	33.1	29.8	26.1	8.2	28.8
Moderna	Respondent 1	18.4	10.2	17.8	17.7	7.9	11.5
	Respondent 2	35.6	33.1	29.8	26.1	9.5	28.8
AstraZeneca	Respondent 1	22	21.4	26	8.5	14.5	12.2
	Respondent 2	9.1	6.1	15.6	10.6	28.1	8.2
Sinopharm	Respondent 1	13.7	31.8	8.2	39.6	33.1	33
	Respondent 2	8.1	14.1	3.3	16.1	9.2	17.9
Sputnik V	Respondent 1	6.7	6.2	5.2	6.8	24.7	19.6
	Respondent 2	4.7	8.3	7.6	10.6	20.7	8.2
Janssen	Respondent 1	20.9	20.3	25	9.7	14.5	12.2
	Respondent 2	6.9	5.3	14	10.6	24.3	8.2

**Table 5:** Sensitivity analysis for data from respondent 1.

Criterion	Vaccines Alternatives	Pfizer-BioNTech	Moderna	AstraZeneca	Sinopharm	Sputnik V	Janssen
	Original Percentage of Relative Weights for Vaccine Selection (from Figure 1)	15.58	15.65	22.38	17.79	7.04	21.56
Availability	Percentage of Relative Weights (When increased by a value of 1)	15.6	15.71	22.41	17.66	7.01	21.61
	Percentage Change	0.13	0.38	0.13	0.73	0.43	0.23
	Percentage of Relative Weights (When decreased by a value of 1)	15.59	15.62	22.23	18.04	7.09	21.43
	Percentage Change	0.06	0.19	0.67	1.41	0.71	0.6
Formula	Percentage of Relative Weights (When increased by a value of 1)	15.45	15.52	22.42	18.06	6.97	21.58
	Percentage Change	0.83	0.83	0.18	1.52	0.99	0.09
	Percentage of Relative Weights (When decreased by a value of 1)	15.72	15.8	22.32	17.52	7.13	21.51
	Percentage Change	0.9	0.96	0.27	1.52	1.28	0.23
Efficacy	Percentage of Relative Weights (When increased by a value of 1	15.73	15.78	22.6	17.21	6.92	21.76
	Percentage Change	0.96	0.83	0.98	3.26	1.7	0.93
	Percentage of Relative Weights (When decreased by a value of 1)	15.42	15.49	22.14	18.46	7.16	21.33
	Percentage Change	1.03	1.02	1.07	3.77	1.7	1.07
Side Effects	Percentage of Relative Weights (When increased by a value of 1)	15.56	15.63	22.39	17.76	7.1	21.56
	Percentage Change	0.13	0.13	0.04	0.17	0.85	0
	Percentage of Relative Weights (When decreased by a value of 1)	15.59	15.66	22.34	17.79	7.09	21.53
	Percentage Change	0.06	0.06	0.18	0	0.71	0.14
Cost	Percentage of Relative Weights (When increased by a value of 1)	15.62	15.67	22.34	17.84	7	21.53
	Percentage Change	0.26	0.13	0.18	0.28	0.57	0.14
	Percentage of Relative Weights (When decreased by a value of 1)	15.53	15.62	22.42	17.73	7.11	21.59
	Percentage Change	0.32	0.19	0.18	0.34	0.99	0.14
Host Factors	Percentage of Relative Weights (When increased by a value of 1)	15.61	15.68	22.33	17.88	6.97	21.53
	Percentage Change	0.19	0.19	0.22	0.51	0.99	0.14
	Percentage of Relative Weights (When decreased by a value of 1)	15.58	15.64	22.34	17.83	7.09	21.52
	Percentage Change	0	0.06	0.18	0.22	0.71	0.19

## Data Availability

All relevant data are included within the manuscript.
